# Perceived Trust and Professional Identity Threat in AI-Based Clinical Decision Support Systems: Scenario-Based Experimental Study on AI Process Design Features

**DOI:** 10.2196/64266

**Published:** 2025-03-26

**Authors:** Sophia Ackerhans, Kai Wehkamp, Rainer Petzina, Daniel Dumitrescu, Carsten Schultz

**Affiliations:** 1 Kiel Institute of Responsible Innovation University of Kiel Kiel Germany; 2 Medical School Hamburg Hamburg Germany; 3 Clinic for General and Interventional Cardiology/Angiology Heart and Diabetes Center North Rhine-Westphalia Ruhr-University Bochum, Medical Faculty OWL (University Bielefeld) Bad Oeynhausen Germany

**Keywords:** artificial intelligence, clinical decision support systems, explainable artificial intelligence, professional identity threat, health care, physicians, perceptions, professional identity

## Abstract

**Background:**

Artificial intelligence (AI)–based systems in medicine like clinical decision support systems (CDSSs) have shown promising results in health care, sometimes outperforming human specialists. However, the integration of AI may challenge medical professionals’ identities and lead to limited trust in technology, resulting in health care professionals rejecting AI-based systems.

**Objective:**

This study aims to explore the impact of AI process design features on physicians’ trust in the AI solution and on perceived threats to their professional identity. These design features involve the explainability of AI-based CDSS decision outcomes, the integration depth of the AI-generated advice into the clinical workflow, and the physician’s accountability for the AI system-induced medical decisions.

**Methods:**

We conducted a 3-factorial web-based between-subject scenario-based experiment with 292 medical students in their medical training and experienced physicians across different specialties. The participants were presented with an AI-based CDSS for sepsis prediction and prevention for use in a hospital. Each participant was given a scenario in which the 3 design features of the AI-based CDSS were manipulated in a 2×2×2 factorial design. SPSS PROCESS (IBM Corp) macro was used for hypothesis testing.

**Results:**

The results suggest that the explainability of the AI-based CDSS was positively associated with both trust in the AI system (β=.508; *P<*.001) and professional identity threat perceptions (β=.351; *P*=.02). Trust in the AI system was found to be negatively related to professional identity threat perceptions (β=–.138; *P*=.047), indicating a partially mediated effect on professional identity threat through trust. Deep integration of AI-generated advice into the clinical workflow was positively associated with trust in the system (β=.262; *P*=.009). The accountability of the AI-based decisions, that is, the system required a signature, was found to be positively associated with professional identity threat perceptions among the respondents (β=.339; *P*=.004).

**Conclusions:**

Our research highlights the role of process design features of AI systems used in medicine in shaping professional identity perceptions, mediated through increased trust in AI. An explainable AI-based CDSS and an AI-generated system advice, which is deeply integrated into the clinical workflow, reinforce trust, thereby mitigating perceived professional identity threats. However, explainable AI and individual accountability of the system directly exacerbate threat perceptions. Our findings illustrate the complex nature of the behavioral patterns of AI in health care and have broader implications for supporting the implementation of AI-based CDSSs in a context where AI systems may impact professional identity.

## Introduction

### Background

Research in the field of health care innovation has begun to examine the impact of artificial intelligence (AI) on human-cognitive structures and processes of medical decision-making and problem-solving [1,2]. AI enables machines to process and analyze vast datasets independently [3], improving clinical outcomes in areas such as sepsis detection and error reduction [4,5]. AI-based clinical decision support systems (CDSSs) can enhance patient care while reducing costs and errors [6]. However, successful implementation requires acceptance by health care professionals, which is often hindered by skepticism, distrust, and perceived threats to professional identity [7,8].

Process design features, such as explainability and reliability, play a crucial role in shaping trust in AI-based CDSSs and may influence physicians’ professional identity [8-11]. While prior research has explored the relationship between trust and technology acceptance [12,13], the impact of these design features on professional identity threats remains underexplored. This study investigates how process design features influence physicians’ trust in AI-based CDSSs and how this trust relates to professional identity threats, providing insights to support AI implementation in health care.

### Conceptual Framework

Early research on technology adoption in organizations primarily focused on how users perceive and respond to technical aspects. For instance, the Technology Acceptance Model [14] delineates the key determinants shaping users’ attitudes and intentions toward adopting new workplace technology. Recent studies have scrutinized technology adoption from the perspective of trust formation [12,15,16], revealing that a lack of trust in technology can lead to its misuse and nonadoption of technology [15]. For example, initial trust in technology positively mediates the relationship between technology-related factors (such as performance expectancy, effort expectancy, and task complexity) and behavioral intentions [16,17]. Trust denotes the degree to which an individual is willing to believe in or be vulnerable to the actions of another party [18]. This is particularly relevant in situations characterized by risk or uncertainty, where individual behavior can lead to loss or harm [19]. Importantly, this description extends trust beyond human interaction to encompass technology, including AI [20]. This study focuses on the user’s trust in the AI-based technology, rather than interpersonal trust (trust between individuals) or social trust (trust in an institution) [21]. Trust is a crucial prerequisite for physicians in adopting AI [22], as AI is perceived as risky due to the complexity and unpredictability of its behavior [11]. Thus, the formation of trust in an AI-based system among physicians is influenced by the AI system’s representation and tangibility, that is how the underlying rationale of AI tools’ decision outcomes are presented to the user [12]. Consequently, the representation of AI functionalities is expected to play a pivotal role in fostering trust in AI among physicians.

IT systems that disrupt traditional work routines often meet with particular resistance. This defensiveness originates, among other things, from the potential threat to autonomous work processes and traditional routines [23]. Studies have emphasized the relevance of a perceived threat or enhancement posed by new technologies to professional identities, underscoring the importance of sociocognitive elements related to human expertise, including hierarchy, status, autonomy, control, and legitimacy, particularly in knowledge-intensive organizations [23-26].

Professional identity pertains to how individuals define and distinguish themselves in their professional roles [27]. Medical professionals exhibit a particularly strong attachment to their social group and professional identity, which evolves through rigorous socialization during education and the acquisition of extensive expertise through practical experience [28,29]. Health care professionals often have the autonomy to make treatment decisions, intensifying their motivation and dedication and reinforcing their identification with the medical field [30]. Disruptions to existing workflows will be perceived as threats to health professionals’ identity if they restrict autonomy in terms of decision-making and sovereignty over medical knowledge [8].

A recent literature review on the implementation challenges of CDSSs showed that a CDSS transparency in elucidating decision outcomes (the “explainable” AI), integration depth of the AI-generated system advice into the clinical workflow, and security- and privacy-related mechanisms such as system-induced individual accountability frame professional identity threats and enhancements among physicians [10]. These 3 process design features of AI-based systems may have positive and negative behavioral consequences.

The AI’s ability to analyze vast amounts of data and transparently present the outcome of the analysis can complement physicians’ expertise and strengthen their professional identity, by facilitating more informed decision-making and affirming physicians in their perceived efficiency and effectiveness. However, as the medical environment in which AI is used is usually complex and the behavior of AI is not deterministic, the opaque, multilayered process of AI decision-making is difficult to predict, and the underlying logic of the decision may be poorly understood [11,30]. The risk of overreliance on AI may lead to a perceived erosion of traditional medical expertise. Physicians may associate AI recommendations with a devaluation of their professional judgment [7].

AI-based systems that integrate seamlessly into clinical workflows can enhance the efficiency and productivity of clinicians, allowing them to focus more on patient care. This can strengthen their trust in the IT system [31]. However, AI systems that provide detailed behavioral advice, which closely resemble physicians’ established decision-making parameters, can disrupt the intrinsic rhythm of medical practice. Such disturbances may foster a perception of diminished autonomy among physicians that could potentially compromise the physicians’ professional identity [10].

In addition, the use of digital signatures and the monitoring of user credentials play a critical role in safeguarding patient information and maintaining trust in the health care system [10,32]. The requirement for user signatures on treatment decisions ensures that actions affected by the AI-based CDSS are traceable to specific, authorized users, validating the technologically derived information, and reinforcing the confidentiality and integrity of patient data. The tracking of user credentials and actions within AI-based CDSS underscores the responsibility of physicians in making informed, ethical treatment decisions, which reinforces their professional identity as accountable caretakers of patient health [32]. However, these systems may on the other hand negatively influence the professional identity and role of physicians by embedding accountability and increasing monitoring of diagnostic and therapeutic decisions by clinical managers and external agents such as government bodies and health insurance [8]. Electronic monitoring facilitates the regulation and control of both processes and outcomes. This technological oversight grants clinical managers and regulators the capability to impose a form of direct governance over health care professionals. It effectively binds physicians to a self-regulatory framework, which is less predicated on the active enforcement of control than on the perceived omnipresence of surveillance [33]. Furthermore, detailed tracking of decisions and actions can be used in legal situations, increasing the liability risks for medical professionals. This might lead to more defensive behavior, where professionals take extra precautions, not out of clinical necessity, but to protect against potential legal repercussions [7].

**Figure 1:**
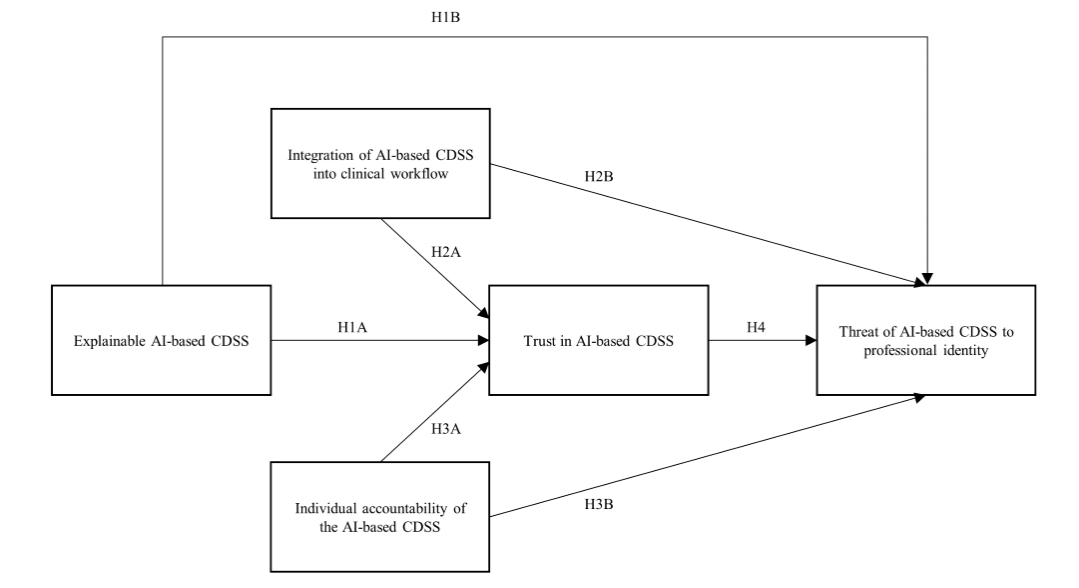
The proposed research framework for analyzing the impact of AI process design features on perceived professional identity threat. AI: artificial intelligence; CDSS: clinical decision support system; H1A: hypothesis 1A; H1B: hypothesis 1B; H2A: hypothesis 2A; H2B: hypothesis 2B; H3A: hypothesis 3A; H3B: hypothesis 3B; H4, hypothesis 4.

In the subsequent section, we formulate hypotheses regarding the role of trust in AI-based CDSSs and the potential threat or enhancement to the professional identity of health care professionals stemming from the use of AI-based CDSSs. In this context, we investigate the impact of AI explainability, depth of integration of the AI-generated system advice into the clinical workflow, and system-induced individual accountability. Figure 1 provides a visual representation of our proposed research model.

#### Hypothesis 1A: The Explainability of an AI-Based CDSS Has a Positive Effect on Trust for Health Care Professionals in the Decision Outcomes of Such Systems

Explainable AI represents a crucial component in building trust in AI-based technologies by ensuring transparency in the system’s operational principles [12,34]. Such a system empowers users to understand the system’s internal operations and decision rationale. For instance, it can illustrate how inputs are mathematically transformed into outputs [35]. Various levels of explainability can be implemented, including the presentation of varying weightings of data leading to AI decisions [36]. Numerous studies highlight the importance of explainable models in health care, enabling health care professionals to trust AI-generated outcomes [37,38]. Consequently, we propose that the transparent display of the data used and the mathematical basis of decision outcomes in an explainable AI-based CDSS fosters higher levels of trust.

#### Hypothesis 1B: The Explainability of an AI-Based CDSS Has a Positive Effect on Physicians’ Professional Identity Threat

Nevertheless, explainable AI-based CDSSs may, in contrast, pose a potential threat to medical authority and the role of physicians [7,8]. As the potential of the AI system becomes transparent to health care professionals, they are more likely to believe in a shift in hierarchy and an erosion of physicians’ autonomy and status as a result of AI systems [8,26,39]. In scenarios where AI partially supersedes a physician’s expertise, particularly in high-risk situations, it exacerbates their sense of loss of control and status [7], which can induce stress as their professional decision-making control and autonomy may be undermined [40]. An explainable AI-based CDSS may as such be perceived as a threat to their established roles and professional standing.

#### Hypothesis 2A: An AI-Based CDSS That Is Deeply Integrated Into the Clinical Workflow Has a Positive Effect on Physicians’ Trust in the System

Evaluating the AI-based CDSSs’ fit into the clinical workflow is crucial because users’ trust in AI varies with the expected efficacy of the technical system [41]. For instance, physicians often disregard AI recommendations that are considered unnecessary, potentially missing out on relevant decision-enhancing benefits [7,42]. Detailed clinical treatment recommendations, especially for high-risk events or complex tasks, can reinforce confidence in the system by reducing uncertainty through the clinical information provision [30,34,43]. Consequently, the degree of trust in the technology’s effectiveness is a result of users’ perception of its ability to offer sufficient, efficient, and prompt assistance.

#### Hypothesis 2B: An AI-Based CDSS That Is Deeply Integrated Into the Clinical Workflow Has a Positive Effect on Physicians’ Perceived Professional Identity Threat

Physicians’ strong identification with their profession [28] may make them skeptical of AI-driven system advice, especially in situations where the AI task undermines their own clinical workflow and tasks [30,44]. Physicians may fear losing control over medical decisions if they are compelled to follow the system’s advice [7]. Integrated AI-based systems with automated advice, which closely mirror established human tasks, may disrupt current established processes. Moreover, explicit advice on expected behavior may diminish professional authority and autonomy, eroding individual control. When systems control actions, the scope for human professional judgment is limited, impacting the ability to provide proficient recommendations. This situation can lead to a sense of redundancy [44].

#### Hypothesis 3A: An AI-Based CDSS That Induces Individual Accountability Has a Negative Effect on Physicians’ Trust in the AI-Based CDSS

A mandatory use of AI-generated feedback, aimed at enhancing human-AI collaboration, can inadvertently bring attention to managerial monitoring and tracking, potentially leading to concerns about excessive control and distrust. For instance, demanding health care professionals to approve AI system decisions can be seen as invasive and indicative of a lack of managerial discretion, which may undermine both trust in the system and compliance [45]. Similar issues have arisen in contexts like Uber (Uber Technologies Inc), where continuous tracking eroded drivers’ autonomy and trust [46]. Thomas et al [47] investigated barriers to adopting e-prescribing and found that requiring health care professional signatures on prescriptions initially caused frustration and a preference for paper-based prescriptions. However, over time, the use of signatures became less of a barrier. Still, constant signature requirements can increase ambiguity about treatment responsibility, leading to reduced trust in the system. Physician signatures for validating the AI system decisions make them cocreators of the AI-based advice, increasing risk awareness and responsibility for the IT system’s actions, and as such decreasing system trust.

#### Hypothesis 3B: An AI-Based CDSS That Induces Individual Accountability Has a Positive Effect on the Physicians’ Perceived Threat to Professional Identity

Active endorsement of AI-based CDSS decisions can lead to an increased perceived reliance of health care professionals on the system. This perceived dependency diminishes their sense of autonomous decision-making and intensifies the pressure on physicians to integrate AI results into their professional judgment [30]. This pressure to endorse and validate AI-based decisions raises questions about the distribution of responsibility and accountability, reducing an individual’s ability to influence a decision [48]. This kind of AI can infringe physicians’ autonomy and control as they have to justify AI-based CDSS decisions, despite a lack of knowledge about the data quality and accuracy of the system [44]. The traceability provided by signatures raises concerns about the benefits and risks of AI systems, potentially jeopardizing a physician's reputation as a medical services provider [49].

#### Hypothesis 4: Trust in an AI-Based CDSS Mediates the Relationship Between an Explainable AI-Based CDSS and Physicians’ Perception of Threat to Their Professional Identity

Finally, we anticipate a mediated relationship between the analyzed process design features of AI systems and their influence on the perception of threats to professional identity via trust. Empirical research has nuanced human trust in AI into the dimensions of cognitive and affective trust [50]. Cognitive trust in AI, rooted in rational evaluation and reliability, and affective trust, driven by emotional and interpersonal connections, both emerge as key facets that influence the acceptance and use of AI [11]. For cognitive trust, AI reliability and explainability have been identified as pivotal, as the degree to which the functionality and decision-making processes of the AI are understandable to users is crucial for building trust. Affective trust, on the other hand, is based on affective bonds and feelings toward AI technology rather than rational evaluations of its capabilities. The degree to which AI demonstrates behaviors that promote perceived closeness to actual medical tasks can foster emotional bonds between users and AI, enhancing trust on an emotional level [11]. Given the relationship between the use of an AI-based CDSS and threat to professional identity, it is reasonable to assume that physicians’ cognitive and affective trust in explainable AI-based CDSSs can reduce their perception of threat to their professional identity.

## Methods

### Study Design

Scenario-based experimental surveys present a valuable approach to quantitative research by integrating the strengths of traditional surveys and experimental designs. This method enhances external validity through the representativeness and multivariate measurement capabilities of surveys, while simultaneously improving internal validity through controlled, experimental interventions. By addressing the limitations of both approaches—namely, the multicollinearity and passive measurement inherent in surveys, and the lack of representativeness and simplified settings typical of experiments—scenario-based studies offer a comprehensive and rigorous method for investigating respondents' beliefs, attitudes, and judgments [51].

### Sample and Data Collection

The study was conducted with German medical students, physicians, and nursing professionals. The study sample was composed primarily of German medical students in the second (clinical) part of the course study program, as they represent future users of AI-based risk prediction systems and are at a critical stage of developing their professional identity. During their education, medical students gain professional experience by interacting directly with patients under supervision, allowing them to apply theoretical knowledge to real-world clinical scenarios. This hands-on training is essential for developing diagnostic skills, clinical decision-making, and effective patient communication. Medical students undergo continuous practical training in university clinics, teaching hospitals, or other medical centers. In addition, they are required to complete a 3-month internship in a hospital before starting their medical study program or during nonteaching periods of the preclinical stage, which familiarizes them with hospital operations and clinical procedures. Furthermore, a 4-month clinical traineeship is mandatory during the transition between the preclinical and clinical stages of medical education. These structured experiences progressively immerse students in hospital environments, allowing them to interact with diverse physicians and develop their professional identities. Research supports the idea that medical students’ professional identity evolves rapidly during their education. Niemi [52] notes that critical moments for identity development occur at the start of professional training, as students begin to conceptualize themselves as future physicians. Similarly, Pitkala and Mantyranta [53] found that while medical students initially lacked confidence, their interactions with patients during clinical placements increased their sense of credibility and comfort by the end of their first year. Madill and Latchford [54] further observed that students’ identity progression during their first year of training was marked by growing competence and dedication. Advanced medical students, therefore, are well-positioned to recognize and articulate perceptions of professional identity threats.

For the study, 8 different scenarios of a fictitious AI-based CDSS were developed, which showed the user the risk of developing sepsis within the next 48 hours. Sepsis is a complex infectious disease that can develop from any focus of infection. It can be caused by viruses, bacteria, fungi, or parasites and can affect patients of all ages [55]. According to an analysis of Germany-wide Diagnosis Related Groups statistics, there was an incidence of 158 patients with sepsis per 100,000 inhabitants in Germany in 2015. The proportion of sepsis patients among all hospital patients was 0.7%. 53.8% of patients with hospital-acquired sepsis were treated in intensive care units and 41.7% died in hospital [56]. In addition to the lack of improvement in treatment standards and measures to reduce hospital-acquired infections, this high mortality rate is attributed to a lack of quality initiatives for early detection [57], which illustrates the high relevance of AI-supported CDSSs for sepsis risk prediction.

Five physicians from a university hospital in northern Germany evaluated the scenario-based web-based survey during a pretest to ensure that all 3 factors studied were successfully manipulated and that participants could distinguish between them. Participants were familiar with the topic of sepsis, as this condition is considered one of the most common preventable causes of death and requires timely antibiotic therapy, which largely depends on the physician’s judgment if an AI-based CDSS is not used. Manipulation checks were conducted to ensure that participants understood the manipulation of the scenario. It was also checked whether the respondents answered the survey seriously (seriousness check).

Participants were recruited via paper and digital flyers distributed in senior medical lectures at medical faculties in northern Germany. In addition, participants were recruited via social media and newsletters from medical faculties at German universities. Participation in the study was voluntary, and participants could access the web-based survey via a link and QR code provided on the recruitment flyers. Before the participants could access the questionnaire, they had to give their informed consent to participate. Participants in the study were able to view information about the duration of the survey and data collection (location and duration of data storage, investigator, and purpose of the study) via a link to a pop-up on the first page of the survey before giving their consent. The data was collected between the end of 2022 and summer 2023. Of the 673 people who clicked on the QR code or link for the survey, 333 (49.48%) participants accessed the survey. We received 292 completed responses, which we used for analysis.

### Design and Manipulations

In a 2×2×2 factorial design, 3 factors were manipulated: AI-based CDSS decision explainability (explained vs not explained), integration depth of AI-based CDSS advice into the clinical workflow (detailed vs no detailed clinical treatment advice), and system-induced accountability of a physician in relation to the AI-based CDSS advice (system required a signature vs no signature). These manipulations were designed to produce 8 different treatments (Table 1). All participants were given a scenario of AI Med Predict, a fictitious AI-based CDSS. We modified the wording of the scenarios while keeping the content consistent to exclude other interpretations. The participants were randomly divided into 8 groups, each exposed to one of the treatments (between-subject design). A minimum size of 23 in each group was calculated with an effect size of 0.85, an α of .05, and a statistical power of 0.8 using the statistical software G*Power. Thus, the cell sizes for the 8 treatments were large enough for this exploratory study (n_1_=29; n_2_=44; n_3_=41; n_4_=45; n_5_=31; n_6_=36; n_7_=42; and n_8_=24) [58,59].

We developed a hypothetical AI-based CDSS tool for sepsis risk prediction to operationalize AI-based CDSS decision explainability, depth of integration of AI-based CDSS advice into the clinical workflow, and system-induced individual accountability. The simulated CDSS, set in a fictional ward view, displayed the patient’s name, age, case number, primary diagnosis, room and bed, progress remark, and estimated sepsis risk score in percent. Subsequently, the respondent has seen a scenario involving a hypothetical patient with a high-risk prognosis for sepsis within 48 hours.

The explainability of the AI-based CDSS decision was altered through 2 treatments. If the user received a detailed explanation of the AI-based CDSS decision that included the patient data used in the risk analysis calculation, such as preexisting conditions, recent procedures, vital signs, laboratory values, or age, we coded the variable as 1. If the user did not receive an explanation of the estimated sepsis risk score, we coded the variable as 0. The integration depth of AI-generated advice into the clinical workflow was coded as 1 if the system specified preventive treatment procedures, such as catheter change and microbiological investigations. The respondent was also provided with a link to the German sepsis prevention, diagnostic, treatment, and aftercare decision guidelines [60]. If the system made an unspecific call for preventive activities, the variable was coded as 0. System-induced individual accountability was coded as 1 if the system hypothetically sought a signature from the respondent to acknowledge the risk prediction and to confirm the planned preventative actions. If the system instructed the user to examine another subject without requiring a signature, the variable was coded as 0.

To ensure treatment efficacy, we used manipulation checks. Specifically, the respondents were asked to evaluate the explainability of the AI-based CDSS decision (eg, whether AI Med Predict for risk prediction of sepsis shows them the composition or explanation of the risk value), the depth of integration of AI-based CDSS advice into the clinical workflow (eg, whether AI Med Predict for risk prediction of sepsis gives them an exact recommended course of action including a link to treatment guideline) regarding the measures they should initiate for the patient), and system-induced individual accountability for the decision outcome of the AI-based CDSS (eg, whether AI Med Predict for risk prediction of sepsis finally prompts them for their signature). Only participants were included in the dataset, which have successfully passed the manipulation check. On average, the survey completion time was 15 minutes 16 seconds.

**Table 1:** Structure of the 2×2×2 scenario-based experiment design with 8 scenarios.

Scenario	Explainability of AI^a^-based CDSS^b^ decision			Integration depth of AI-based CDSS advice into the clinical workflow			Individual accountability induced by AI-based CDSS				
	Explained	Not explained	Detailed treatment advice		No detailed treatment advice	Signature required		No signature required			
1		✓			✓			✓			
2	✓				✓			✓			
3		✓	✓					✓			
4	✓		✓					✓			
5		✓			✓	✓					
6	✓				✓	✓					
7		✓	✓			✓					
8	✓		✓			✓					

^a^AI: artificial intelligence.

^b^CDSS: clinical decision support system.

### Measurement Scales

All question items were measured using a 5-point Likert scale from “strongly disagree” to “strongly agree” (Multimedia Appendix 1 lists the measurement items in detail). Professional identity threat was measured by a 6-item scale adopted from Walter and Lopez [8]. Sample items include “Using AI Med Predict to predict risk of sepsis reduces my control over clinical decisions.” Trust in AI-based CDSSs was measured by adapting the 8-item scale developed by Hoffman et al [34]. An example item is “I have confidence in the AI Med Predict system for risk prediction of sepsis. I have the feeling that it works well.” Because the 2 scales represent relatively recent additions and include self-developed items, we conducted an exploratory factor analysis (EFA) to assess their construct validity [61]. For the scales we used, a principal component analysis with varimax rotation was performed. The EFA enabled all items to load on any factor, with the number of factors being defined by an eigenvalue larger than 1.00 [62]. As reported in Multimedia Appendix 2, this analysis yielded 2 factors. The first is composed of items 1, 2, 3, and 5 of the professional identity threat scale adapted from [8]. The second factor includes the first 4 items of the AI trust scale proposed by [34]. Any remaining items that failed to load on their respective scales were subsequently eliminated. Despite this, the scales appear cohesive enough for analysis. The scales for professional identity threat and trust in technology demonstrated reliability, with Cronbach α values of 0.83 and 0.81, respectively [63].

Personal innovativeness with technology was examined as a covariate using a scale, titled the same as Agarwal and Prasad [64], which measures the respondent’s willingness to explore new information technology. EFA was used to evaluate the personal innovativeness scale's component structure [61]. We found a single-factor solution for personal innovativeness with a technology scale and a satisfactory Cronbach α (0.86), indicating excellent construct validity and reliability of these measures [63,64].

### Ethical Considerations

Ethical approval was granted by the Central Ethics Committee of Kiel University (ZEK-19/22). Informed consent was obtained from all participants prior to participation in the scenario-based experiment. The confidentiality of the participants was maintained at all times. The participants received no compensation for their participation. In return for participating in the survey, we promised an earmarked donation per completed response to a medical charity in the survey.

## Results

### Descriptive Statistics

A total of 333 responses were collected. Respondents who indicated that their education or employment was not related to the health care field were excluded, as their experiences may not be relevant to the research question (n=16, 4.8%). Manipulation checks were included to verify whether participants properly understood and engaged in experimental conditions. Participants who failed these checks were excluded to maintain data integrity (n=16, 4.8%). In addition, seriousness checks were used to ensure participants provided thoughtful responses. Those who failed these checks, typically by answering in a way that indicated random or inattentive participation, were excluded (n=9, 2.7%). This resulted in a final sample of 292 (87.7%) participants for data analysis. 278 (95.2%) were medical students, 8 (2.7%) participants were physicians across different specialties, and 6 (2.1%) were nursing professionals (eg, intensive care nurses). The participants’ average age was assessed using a categorical scale, where 1=18 years or younger and 7=60 years or older. The mean score for age was 2.53 (SD 0.75), indicating that the typical participant in the sample was approximately 24-25 years of age, corresponding to the mid-20s age range. 70% of the participants were female (Table 2). On average, the medical students attended their seventh semester and 74.1% had 3 or more years of bedside experience. 75% of the physicians and 83.3% of the nursing professionals were younger than 30 years and were still active in medical specialist training. 57.2% of the respondents stated that they lived in Schleswig-Holstein or Hamburg, 9.6% in Lower Saxony, 9.3% in Baden-Wuerttemberg, and 8.9% in Bavaria. The complete demographic characteristics of the sample are present in Multimedia Appendix 3.

The mean values, SDs, and Pearson correlations for the study variables are displayed in Table 2. The mediator and dependent variables are weakly correlated (trust and professional identity threat: *r*=–0.119; *P=*.042). Explainability of the AI-based CDSS’ decision outcome and trust in an AI-based CDSS are moderately correlated (*r*=0.367; *P*<.001). AI-based system advice (*r*=–0.115; *P=*.049) and accountability of the AI-based CDSS via a system-required signature (*r*=0.122; *P=*.037) are weakly correlated with professional identity threat.

**Table 2:** Mean values, SDs, and correlation matrix (N=292).

	Mean	SD	1	2	3	4	5	6	7	8
Explainable AI^a^-based CDSS^b^	0.49	0.501	1.00	—^c^	—	—	—	—	—	—
AI-based CDSS system advice	0.48	0.500	–0.117^d^	1.00	—	—	—	—	—	—
AI-based CDSS tracking	0.54	0.499	–0.108	–0.045	1.00	—	—	—	—	—
Trust	3.278	0.655	0.367^e^	0.114	–0.022	1.00	—	—	—	—
Professional identity threat	1.907	0.708	0.034	–0.115^f^	0.122^g^	–0.119^h^	1.00	—	—	—
Personal innovativeness	3.463	0.799	0.069	–0.016	–0.005	0.074	–0.103	1.00	—	—
Age (1-7 scale)^i^	2.50	0.780	–0.009	0.137^j^	–0.036	–0.104	–0.027	–0.077	1.00	—
Sex (1: female)	0.70	0.58	0.039	–0.064	0.006	0.133^k^	–0.030	–0.300^l^	–0.089	1.00

^a^ AI: artificial intelligence.

^b^CDSS: clinical decision support system.

^c^Not applicable.

^d^*P*=.045.

^e^*P<*.001.

^f^*P=*.049.

^g^*P=*.04.

^h^*P=*.042.

^i^Participants reported their age using a categorical scale: 1=18 years or younger, 2=19-24 years, 3=25-29 years, 4=30-39 years, 5=40-49 years, 6=50-59 years, and 7=older than 60 years.

^j^*P=.*02.

^k^*P=.*02.

^l^*P<*.001.

### Hypothesis Testing

**Figure 2:**
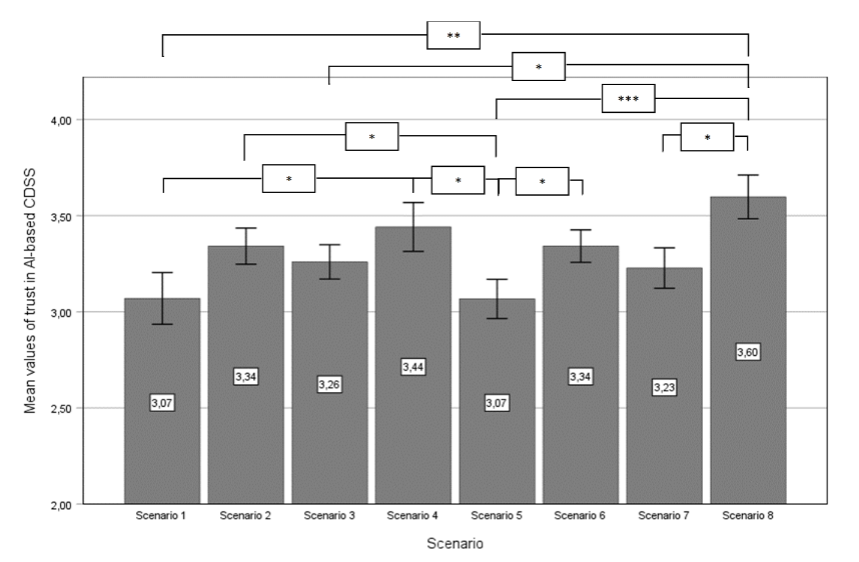
Mean values of variable trust in AI-based CDSS, with pairwise comparisons (post hoc results). AI: artificial intelligence; CDSS: clinical decision support system. *P<.05, **P<.01, ***P<.001.

**Figure 3:**
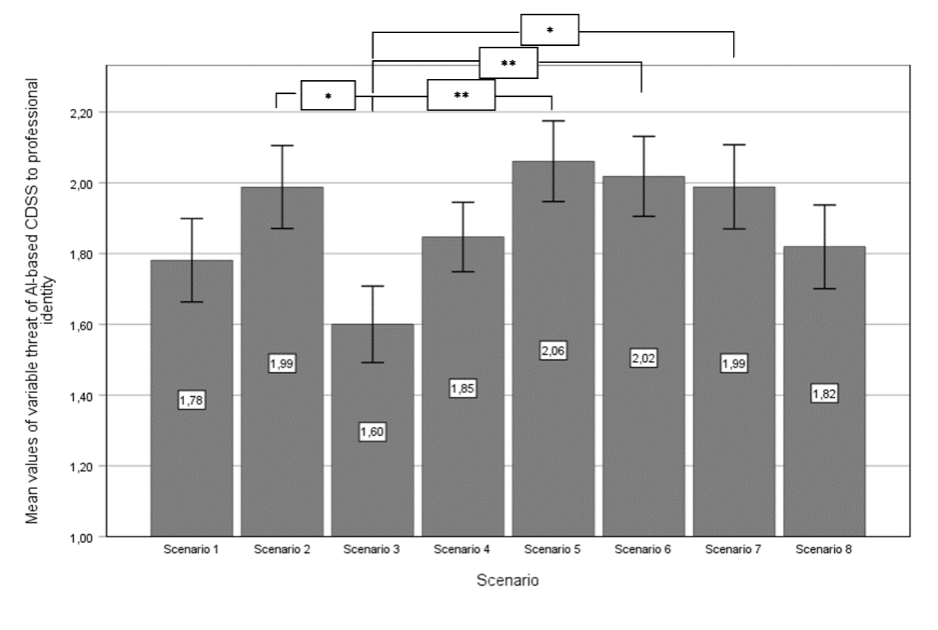
Mean values of variable threat of AI-based CDSS to professional identity, with pairwise comparisons (post hoc results). AI: artificial intelligence; CDSS: clinical decision support system. *P<.05, **P<.01.

First, we conducted a 1-way ANOVA to assess the effects of the 8 differently manipulated scenarios on trust in an AI-based CDSS and the threat of AI-based CDSS to professional identity. The level of trust differed statistically significant for the different scenarios, (*F_7,284_*=2.596; *P*<.01). The level of perceived professional identity threat differed statistically significant on a 10% level for the different scenarios (*F_7,284_*=1.818; *P*<.08). Figure 2 and Figure 3 show the mean values of the 2 variables across the 8 scenarios, along with pairwise comparisons using a post hoc least significant difference test to control for type I error.

The 8 scenarios given in Figures 2 and 3 are as follows. In scenario 1, the AI-based CDSS decision is not explained, the treatment advice is not deeply integrated into the clinical workflow, and there is no individual accountability induced by the system via signature. In scenario 2, the AI-based CDSS decision is explainable; however, the treatment advice remains not deeply integrated into the clinical workflow, and there is no individual accountability induced by the system via signature. Scenario 3 features an AI-based CDSS decision that is not explained, but the treatment advice is deeply integrated into the clinical workflow, while individual accountability is not induced by the system via signature. In scenario 4, the AI-based CDSS decision is explainable, the treatment advice is deeply integrated into the clinical workflow, and no individual accountability is induced by the system via signature. Scenario 5 involves a not explained AI-based CDSS decision with treatment advice that is not deeply integrated into the clinical workflow; however, individual accountability is induced by the system via signature. In scenario 6, the decision is explainable, the treatment advice is not deeply integrated into the clinical workflow, and individual accountability is induced by the system via signature. Scenario 7 presents a not explained AI-based CDSS decision combined with deeply integrated treatment advice in the clinical workflow, and individual accountability is induced by the system via signature. Finally, scenario 8 includes an explainable AI-based CDSS decision, deeply integrated treatment advice within the clinical workflow, and individual accountability is induced by the system via signature. In all scenarios, the error bars represent an SE of 1.

**Figure 4:**
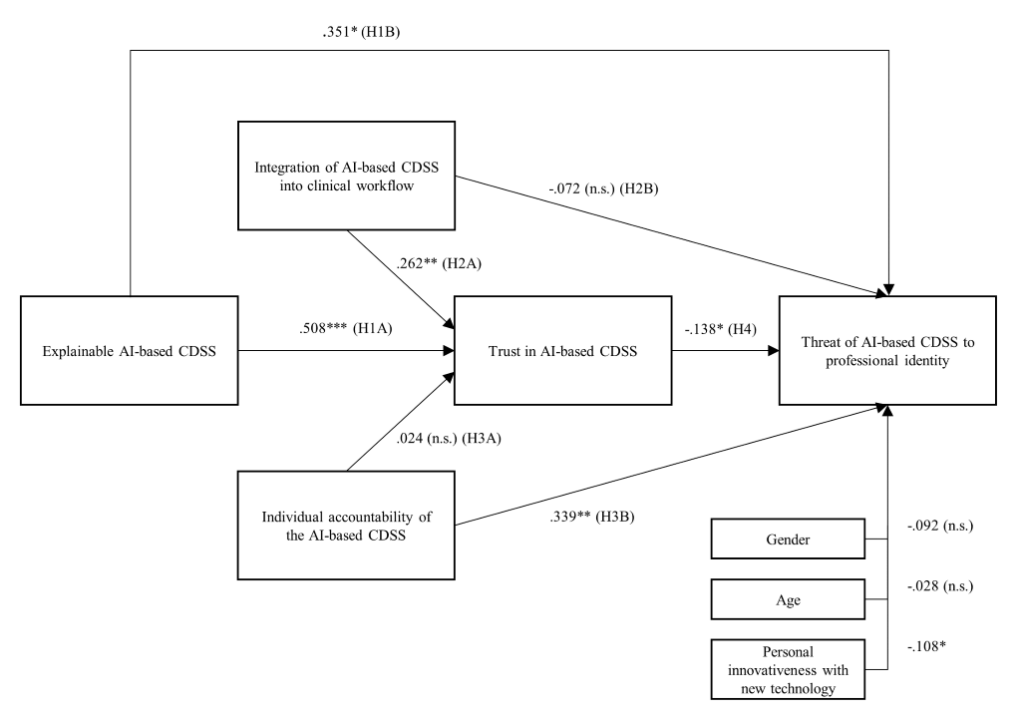
Regression results (N=292). AI: artificial intelligence; CDSS: clinical decision support system; H1A: hypothesis 1A; H1B: hypothesis 1B; H2A: hypothesis 2A; H2B: hypothesis 2B; H3A: hypothesis 3A; H3B: hypothesis 3B; H4, hypothesis 4.

Further, we performed SPSS PROCESS (IBM Corp) macro model 10 to test the proposed hypotheses (Figure 4) [65]. PROCESS is an observed-variable modeling tool that relies on ordinary least squares regression, mediation, or moderation analysis [66]. We carried out bootstrapping to test the indirect effects with a sample size of 5000 and a 95% CI [67]. As suggested by Preacher and Hayes [67], we examined the significance of the effects by checking whether the CIs included 0. As control variables, we considered participants’ age, sex, and personal innovativeness in relation to new technologies. The model explains 7.2% of the variance of the dependent variable, professional identity threat (adjusted *R^2^*=0.072; *F_9,282_*=2.439; *P*=.01). We found a significant positive association between AI-based CDSS explainability and trust in such a system (β=.508; *P*<.001). This finding provides support for hypothesis 1A. Furthermore, it was shown that the level of trust in an AI-based CDSS has a negative relationship with a perceived threat to professional identity (β=–.138; *P*=.047). The results further demonstrated that the indirect effect of the explainability of an AI-based CDSS on professional identity threat via trust in an AI-based CDSS was significant in all treatment manipulations. While holding the other 2 process design features constant, the indirect effect of the explainability of AI via trust was a×b=–.073 (95% CI –0.145 to –0.009). In addition, we found a significant positive direct association between the explainability of an AI-based CDSS and professional identity threat (β=.351; *P*=.02), providing support for hypothesis 1B. Thus, the impact of explainable AI-driven CDSS on the perception of professional identity threat is found to be partially mediated by the level of trust placed in AI, providing support for hypothesis 4. We found a significant positive association between an AI-based CDSS that is strongly integrated into the clinical workflow and trust in such a system (β=.262; *P*=.009), providing support for hypothesis 2A. Further, the direct effect of an AI-based CDSS inducing individual accountability on the perceived threat to professional identity was significant (β=.339; *P*=.004), which supports hypothesis 3B. Finally, sex (1: female) significantly influences trust in an AI-based CDSS (β=.208; *P*=.01) while personal innovativeness significantly influences the professional identity threat posed by an AI-based CDSS (β=–.108; *P*=.046; Figure 4). At the same time, the direct influence of a strongly integrated AI-based CDSS into the clinical workflow on a perceived threat to professional identity is not significant. Similarly, a system inducing a physician’s accountability does not significantly affect trust in an AI-based CDSS (Table 3).

**Table 3:** Path analysis results with coefficients, *P* values, and 95% CIs (N=292).

Hypothesis and path	Coefficients (β)	*P* value	95% CI
H1A^a^: AI^b^-based CDSS^c^ explainability → trust in the AI-based CDSS	.508	<.001	0.258 to 0.758
H1B^d^: AI-based CDSS explainability → perceived threat of AI-based CDSS to professional identity	.351	.02	0.052 to 0.651
H2A^e^: Integration of AI-based CDSS advice into the clinical workflow → trust in the AI-based CDSS	.262	.009	0.067 to 0456
H2B^f^: Integration of AI-based CDSS advice into the clinical workflow → perceived threat of AI-based CDSS to professional identity	–.072	.538	–0.301 to 0.158
H3A^g^: Individual accountability of the AI-based CDSS → trust in the AI-based CDSS	.024	.81	–0.173 to 0.222
H3B^h^: Individual accountability of the AI-based CDSS → perceived threat of AI-based CDSS to professional identity	.339	.004	0.110 to 0.569
H4^i^: Trust in the AI-based CDSS → perceived threat of AI-based CDSS to professional identity	–.138	.047	–0.275 to –0.002
AI-based CDSS explainability → trust in the AI-based CDSS → perceived threat of AI-based CDSS to professional identity	–.073	—^j^	–0.145 to –0.009

^a^H1A: hypothesis 1A.

^b^AI: artificial intelligence.

^c^CDSS: clinical decision support system.

^d^H1B: hypothesis 1B.

^e^H2A: hypothesis 2A.

^f^H2B: hypothesis 2B

^g^H3A: hypothesis 3A.

^h^H3B: hypothesis 3B.

^i^H4: hypothesis 4.

^j^Not applicable.

A formal test of mediation was performed to analyze, independently of the other variables of influence, whether explainable AI-based CDSS predicts a perceived professional identity threat, and whether this direct path would be mediated by trust in the system. A total effect of an explainable AI-based CDSS on professional identity threat perceptions without involving trust was not observed (β=.065; *P=*.43). However, after incorporating the mediator trust in AI into the model, the explainability of an AI-based CDSS significantly predicted the mediator (β=.465; *P<*.001), which in turn significantly influenced perceptions of professional identity threat (β=–.156; *P<*.05). The direct effect of an explainable AI-based CDSS on professional identity threat perceptions via trust was not significant (β=.138; *P=*.12). Thus, our findings suggest that the relationship between AI-based CDSS explainability and professional identity threat perceptions is partially mediated by the trust in the system (indirect effect a×b=–.073 (95% CI –0.143 to –0.009).

Moreover, we have refined the estimation of the model by including other control variables, namely 5 personality dimensions: conscientiousness, extraversion, agreeableness, emotional stability, and openness to experience [68], as well as health care job tenure, and surgical specialties. Here we found that none of the variables had a significant effect on the threat to professional identity or on the change in path coefficients.

## Discussion

### Principal Results

The present study examines how AI-based CDSS process design features affect health care professionals’ trust in AI and their perceived threat to professional identity. The findings show that decision outcome representations and human-AI interaction mechanisms shape health care professionals' trust and professional identity threat perceptions. Our study revealed that trust in AI partially mediates the influence of an explainable AI-based CDSS and strongly integrated AI-based system on the perceived identity threats among health care professionals. This supports earlier studies on AI explainability and clinical workflow integration, establishing trust in technology [10,34]. While AI decision-making may be challenging to understand in certain instances, providing clarity about the algorithm and AI advisory behavior may help align consumers' expectations with the performance of AI [11]. Our study also found a direct and significant positive effect of explainable AI on the perceived threat to professional identity, a finding that aligns with research on the threat posed by AI in professional work [8]. In this context, AI's enhanced capabilities may raise questions not only about its compatibility with physicians’ expertise and experience but also about its individual consequences. Future research needs to ascertain whether explainable AI undermines the perceived professional identity, whether trust in technology stabilizes through emergent dependability in real-life settings, or if this finding is favored by the controlled laboratory setting.

### Comparison With Prior Work

In research on AI-based CDSSs, the effect of system integration into the clinical workflow on the emergence of professional identity threats has been investigated and considered as evidence of high absorption of individual medical competence [7,26,30]. Our study does not find any significant effect of AI-based system advice that is deeply integrated into the clinical workflow on individual professional identity threat assessments. This might be attributed to the different interpretations individuals have with high information content and medical guidelines. Future research should investigate human biases associated with AI-based advice, such as medical guidelines, not only to gain a deeper understanding of their functioning role in shaping professional identity threat perceptions among health care professionals but also to prevent their misuse or overuse. Furthermore, future AI research should focus on the influence of AI-based systems’ reliability, that is, predicted consistent performance [12], on technological trustworthiness [11]. Another potential area of investigation may be the influence of various representations of AI reliability on threats to professional identity. Furthermore, the ongoing discussion about the ethical responsibility of AI-based CDSSs plays a significant role in this context [12]. Physicians must be able to adhere to the system’s decision without fear of biased data composition.

Previous research on monitoring the behavior of health care professionals has identified it as an important factor for distrust in technology [45]. However, as our research indicates, the relationship between AI system monitoring and trust in AI could be more complex, as a system that induces accountability of the physician does not necessarily result in low trust in the AI system and rejection among health care professionals. Health care professionals may perceive a signing function as micromanagement and distrust it, for example, by misusing it to avoid responsibility and traceability of the AI tool’s judgment. Our findings imply that such system functionalities interfere profoundly with personal physician identity perceptions. The significant positive relationship between an AI-assisted system that is inducing individual accountability and a perceived threat to professional identity demonstrates that agreement to an AI-assisted treatment decision is viewed as invasive and antagonistic to professional competence. Nevertheless, decision support can be seen as a functionality that enhances professional identity as it is followed rather than relying on individual professional competence. Especially in situations where direct consultation with a more experienced physician is not available, the reassurance of an AI-assisted decision and its validation may lead to increased trust in the technology [30]. Future research should explore how incorporating a human decision into an AI-assisted CDSS promotes trust in the technology and the socio-cognitive aspects of such technology adoption in the medical context.

The evaluative mechanisms used in this investigation to determine trust in AI outcomes predominantly address cognitive trust. Yet, empirical evidence underscores the significant role of emotional dimensions, such as affective trust, in the efficacious deployment of AI within the health care sector. It is imperative that forthcoming research delves more deeply into the determinants influencing the emotional aspects of trust in AI and additional factors such as cooperation and reliance in physician-AI relations [11].

Adopting such a human-centric perspective presents a substantial opportunity for interdisciplinary collaboration among scholars interested in the evolution and assimilation of AI technologies. While the bulk of existing studies on the interplay between humans and AI are mainly conducted by experts in cognitive engineering and information systems, the field of organizational research offers a valuable vantage point.

This study offers valuable insights for health care managers as well as developers and implementers of AI-based CDSSs, which should approach AI integration with an understanding of the health care professionals' perspective, factoring in aspects that foster human trust in AI and the relevance and value of specific tasks to the workforce. Consequently, in determining the tasks to delegate to AI, it is critical to evaluate not only the technological capabilities at their disposal but also to consider the human element, including perceived threats or enhancements to their professional identity, the interests and motivations of the employees, and the potential for enhancing their trust and productivity through AI collaboration. Specifically, our study shows that the explanation of AI-based CDSS decision outcomes proves beneficial for enhancing trust in AI among health care professionals. AI implementers and health care executives should focus on presenting AI decisions as recommendations that align with existing clinical workflows and which can be overruled by a human decision, rather than disrupting work processes. Furthermore, whenever feasible, AI-based CDSSs should refrain from querying accountability that exposes personal information about the operator. To resolve liability issues, established standards and legal frameworks should be developed, freeing individual practitioners of the duty of actively validating this information.

### Limitations

This study has several limitations that warrant careful consideration. First, the small sample size constrained the ability to apply certain filtering criteria, thereby limiting the robustness of findings related to the influence of specific variables. A larger sample would facilitate more precise filtering, particularly the exclusion of preclinical medical students whose limited professional experience and socialization may not accurately represent perceptions of threats to professional identity. Moreover, the sample’s composition, with 95% comprising medical students, presents a potential selection bias, as their experiences and perceptions may differ significantly from those of practicing physicians, nurses, or other health care professionals. Consequently, the generalizability of the findings to a broader health care population is limited. Future research should aim to include a more heterogeneous sample of health care professionals to enhance the validity and applicability of the results.

Additionally, the study’s use of scenario-based surveys, while methodologically advantageous in combining elements of both traditional surveys and experimental designs, presents inherent limitations. Despite attempts to balance internal and external validity, scenario-based studies may still fail to fully capture the complexity of real-world clinical settings, thus constraining the external validity and broader applicability of the findings [51]. For instance, laboratory studies indicate that users initially exhibit high levels of trust in AI-based CDSS, which diminishes over time as inconsistencies in risk prediction arise [69]. In contrast, field studies suggest that lower initial trust may increase through sustained interaction [70]. Such discrepancies highlight the difficulty in generalizing findings from controlled experimental settings to real-world environments. Furthermore, variability in respondents’ interpretations of the hypothetical scenarios presented may introduce bias, potentially affecting the reliability of the results [51].

Finally, the study’s focus on an AI-based risk prediction system for sepsis may limit the generalizability of the findings to other clinical conditions or AI applications in different contexts. Future research should examine whether the observed effects are consistent across AI systems designed for other clinical functions to provide a more comprehensive understanding of the factors influencing the adoption and trust of AI in health care settings.

### Conclusions

In this study, we demonstrated how distinct AI process design features such as the explainability of AI-based CDSS decision outcomes, the integration depth of AI-based CDSS advice into the clinical workflow, and higher accountability through enforced signatures shape trust in AI and professional identity among health care professionals. Our research demonstrated the critical role that trust in AI played in fostering professional identity among health care workers. While explainable AI-based CDSS and systems highly integrated into clinical workflows enhanced trust, which supported the compatibility of the AI system with professional identity among health care professionals, explainable AI and enforced accountability led to a perceived professional identity threat. The findings of this study can be applicable to a variety of scenarios where the potential threat to professional identity posed by the rising deployment of AI-based systems played a role, especially in knowledge-intensive settings, where well-established and taken-for-granted behaviors, attitudes, and beliefs are questioned by AI solutions.

## Appendix

Multimedia Appendix 1 Final list of items used for the hypothesis testing.

Multimedia Appendix 2 Exploratory factor analysis.

Multimedia Appendix 3 Sample properties of medical students, physicians, and nursing professionals.

## Abbreviations

AI: artificial intelligence
CDSS: clinical decision support system
EFA: exploratory factor analysis

## Data Availability

Data are available from the corresponding author on reasonable request.
