# Supplementary material for: Perceived Trust and Professional Identity Threat in AI-Based Clinical Decision Support Systems: Scenario-Based Experimental Study on AI Process Design Features
Source: JMIR Form Res. 2025 Mar 26;9:e64266. doi: 10.2196/64266 (PMC11982750; doi:10.2196/64266)
Supplement: Multimedia Appendix 1 [file formative_v9i1e64266_app1.docx]

Multimedia Appendix 1**:** Final list of items used for the hypothesis testing^a^

| Construct | Item |
| --- | --- |
| Personal innovativeness with technology | |
|  | P1: If I heard about a new information technology, I would look for ways to experiment with it.  P2: Among my peers, I am usually the first to try out new information technologies.  P3: In general, I am hesitant to try out new information technologies.  P4: I like to experiment with new information technologies. |
| Threats to professional identity [adapted from 11] | |
|  | I1: Using AI Med Predict to predict risk of sepsis reduces my control over clinical decisions.  I2: Using AI Med Predict to predict risk of sepsis reduces my professional discretion over patient care decisions.  I3: Using AI Med Predict to predict risk of sepsis reduces my control over every step in the patient care process.  I4*: The use of AI Med Predict for sepsis risk prediction allows for increased monitoring of my diagnostic and therapeutic decisions by non-physician providers.  I5: Using AI Med Predict to predict risk of sepsis reduces my control over the allocation of scarce resources.  I6*: I see AI Med Predict for risk prediction of sepsis as beneficial to the medical profession as a whole. (R) |
| Trust in AI [adapted from 15] | |
|  | T1: I have confidence in the AI Med Predict system for risk prediction of sepsis. I have the feeling that it works well.  T2: The results of AI Med Predict on risk prediction of sepsis are very comprehensible.  T3: The AI Med Predict system for risk prediction of sepsis works very reliably.  T4: I feel confident that I am getting the right answers when I use the AI Med Predict sepsis risk prediction system.  T5*: AI Med Predict for risk prediction of sepsis can solve the task better than an inexperienced human user. |

^a^ Items with the identifiers* were removed because of measurement properties.
