# Supplementary material for: Perceived Trust and Professional Identity Threat in AI-Based Clinical Decision Support Systems: Scenario-Based Experimental Study on AI Process Design Features
Source: JMIR Form Res. 2025 Mar 26;9:e64266. doi: 10.2196/64266 (PMC11982750; doi:10.2196/64266)
Supplement: Multimedia Appendix 2 [file formative_v9i1e64266_app2.docx]

Multimedia Appendix 2**:** Exploratory Factor Analysis

| Component: | 1 | 2 | 3 | 4 | 5 | 6 |
| --- | --- | --- | --- | --- | --- | --- |
|  |  |  |  |  |  |  |
| P1 |  | .845 |  |  |  |  |
| P1 |  | .822 |  |  |  |  |
| P3 |  | .839 |  |  |  |  |
| P4 |  | .826 |  |  |  |  |
| I1 | .821 |  |  |  |  |  |
| I2 | .856 |  |  |  |  |  |
| I3 | .820 |  |  |  |  |  |
| **I4** | .322 |  |  |  | .315 |  |
| I5 | .710 |  |  |  |  |  |
| **I6** | .315 |  | -.337 | -.523 |  | -.336 |
| T1 |  |  | .723 |  |  |  |
| T2 |  |  | .755 |  |  |  |
| T3 |  |  | .794 |  |  |  |
| T4 |  |  | .771 |  |  |  |
| **T5** |  |  |  | .530 |  |  |

P, Personal innovativeness with technology; I, Threats to professional identity; T, Trust in AI. All loadings < 0.30 are suppressed. Usage inhibitor items are bolded.
