# Supplementary material for: Perceived Trust and Professional Identity Threat in AI-Based Clinical Decision Support Systems: Scenario-Based Experimental Study on AI Process Design Features
Source: JMIR Form Res. 2025 Mar 26;9:e64266. doi: 10.2196/64266 (PMC11982750; doi:10.2196/64266)
Supplement: Multimedia Appendix 3 [file formative_v9i1e64266_app3.docx]

Multimedia Appendix 3**:** Sample properties of medical students, physicians, and nursing professionals

|  | **Medical students** | **Physicians** | **Nursing professionals** |
| --- | --- | --- | --- |
| **Total sample size, N** | 300 | 10 | 7 |
| **Sample used for data analysis, n (%)** | 278 (92.67) | 8 | 6 |
| **Gender (female), n (%)** | 195 (70.14) | 5 | 5 |
| **Age (years), n (%)** |  |  |  |
| 18-24 | 171 (61.51) |  | 2 |
| 25-29 | 83 (29.86) | 6 | 3 |
| 30-39 | 21 (7.55) |  |  |
| 40-49 | 3 (1.08) |  | 1 |
| 50-59 |  | 2 |  |
| **Semester (average)** | Seventh semester |  |  |
| **Professional experience (years), n (%)** |  |  |  |
| 0-2 | 72 (25.90) | 5 | 1 |
| 3-5 | 106 (38.13) | 1 | 3 |
| 6-8 | 70 (25.18) |  |  |
| 9-11 | 21 (7.55) |  |  |
| > 11 | 9 (3.24) | 2 | 2 |
| **Specified place of residence (German state), by ascending postal code, n (%)** |  |  |  |
| Saxony | 2 (0.72) |  |  |
| Saxony-Anhalt | 2 (0.72) |  |  |
| Thuringia | 2 (0.72) |  |  |
| Berlin | 3 (1.08) |  |  |
| Brandenburg | 3 (1.08) |  |  |
| Mecklenburg-Western Pomerania | 4 (1.44) |  |  |
| Hamburg | 13 (4.68) |  |  |
| Lower Saxony | 27 (9.71) | 1 |  |
| Schleswig-Holstein | 144 (51.80) | 5 | 5 |
| Hesse | 2 (0.72) |  | 1 |
| North Rhine-Westphalia | 21 (7.55) | 1 |  |
| Rhineland-Palatinate | 1 (0.36) |  |  |
| Saarland | 2 (0.72) |  |  |
| Baden-Wuerttemberg | 26 (9.35) | 1 |  |
| Bavaria | 26 (9.35) |  |  |
